# Evidence summary of best practices for prevention and management of postpartum hemorrhage in obstetric clinical practice

**DOI:** 10.3389/fmed.2026.1823590

**Published:** 2026-06-24

**Authors:** Yue Bai, Qing Hu, Jianqing Zhang

**Affiliations:** Yan'an Medical College of Yan'an University, Yan'an, Shanxi, China

**Keywords:** best evidence, evidence synthesis, evidence-based nursing, postpartum hemorrhage, prevention

## Abstract

**Objective:**

To search, evaluate, and synthesize the best available evidence for the prevention of postpartum hemorrhage (PPH). This study summarizes the key recommendations of evidence-based nursing for the prevention and management of PPH in obstetrics, aiming to guide clinical practice.

**Methods:**

The PIPOST framework was employed to comprehensively define the evidence-based question across six dimensions: Population, Intervention, Professionals, Outcome, Setting, and Type of evidence, ensuring scientific rigor and relevance. A top-down systematic search was conducted following the “6S” pyramid evidence model, encompassing authoritative international and domestic resources, including computerized decision support systems, guideline repositories, professional society websites, and major databases. Diverse evidence types, such as clinical decisions, guidelines, and systematic reviews, were included to provide comprehensive coverage of PPH prevention strategies. The quality of the eligible literature was rigorously appraised, and the guidelines were evaluated using the for Appraisal of Guidelines Research, Evaluation, and Education Development II instrument to ensure reliability and validity. Key information related to PPH prevention was then extracted. Finally, the evidence was graded based on quality and reliability to provide a clear foundation for evidence-based practice and assist healthcare professionals in selecting appropriate interventions. Thirteen publications were ultimately included, comprising two guidelines, two expert consensuses, one evidence summary, three nursing and survey studies, one systematic review, and three review articles. Twenty-three best practice recommendations for PPH prevention and management were systematically synthesized across 11 domains: risk assessment, anemia management, health education, mode of delivery selection, oxytocin administration, active management of the third stage of labor, labor control, vital signs monitoring, vaginal blood loss monitoring, uterine contraction monitoring, and psychological care.

**Conclusion:**

Clinical administrators bear significant responsibility and should focus on refining the emergency management protocols for severe PPH. Therefore, it is crucial to vigorously promote the standardization and systematic training of healthcare professionals. Through diverse training formats and specialized courses, the professional competence and practical skills of clinical staff in preventing and managing PPH can be effectively enhanced. This enables more effective handling of PPH cases in clinical practice, potentially reducing its incidence and associated mortality, thereby providing a more robust safeguard for maternal health.

**Significance:**

By systematically searching and synthesizing authoritative domestic and international resources, this study summarizes 23 best practice recommendations for PPH prevention and management. This assists nursing staff in conducting better nursing assessments, diagnoses, and planning, formulating scientific and effective nursing interventions, improving workflows, and addressing existing and potential patient problems, thereby promoting scientific and effective clinical nursing practice.

**Scope of application:**

Applicable to parturients with bleeding tendencies, for the prevention of PPH, and to assist healthcare workers in developing scientific and effective medical and nursing measures.

## Introduction

Postpartum hemorrhage remains the leading cause of maternal mortality worldwide, accounting for approximately 27% of all maternal deaths. This phenomenon is particularly prominent in economically underdeveloped regions (1), indicating that the diagnosis and treatment of postpartum hemorrhage are still generally inadequate on a global scale. Currently, there are certain differences in the definition of postpartum hemorrhage among different countries and regions, mainly in terms of the mode of delivery, cumulative blood loss, and accompanying symptoms. According to the 2023 “Chinese Medical Association Guidelines” and the 10th edition of the “Obstetrics and Gynecology” textbook, postpartum hemorrhage is defined as blood loss ≥500 mL within 24 h after childbirth for vaginal delivery or ≥1,000 mL for cesarean section (2, 3). The American College of Obstetricians and Gynecologists does not differentiate by delivery method and directly defines postpartum hemorrhage as blood loss of ≥1,000 mL (4). The Queensland Postpartum Hemorrhage Guidelines use 500 mL as the diagnostic standard for postpartum hemorrhage, 1,000 mL for severe postpartum hemorrhage, and 2,500 mL for massive postpartum hemorrhage (5). Although there are differences among various academic organizations in the specific definition of postpartum hemorrhage, all consistently agree that blood loss exceeding 1,000 mL constitutes severe postpartum hemorrhage, and all fatal cases of postpartum hemorrhage go through a phase of severe postpartum hemorrhage. Therefore, high-risk factor assessment, timely identification, and active intervention for severe postpartum hemorrhage are core aspects of postpartum hemorrhage management, which can effectively reduce the incidence and mortality of severe complications related to postpartum hemorrhage. Therefore, this study aimed to summarize the best evidence for the prevention and nursing of postpartum hemorrhage by retrieving, evaluating, and integrating relevant research.

## Data and methods

### Problem establishment

The PIPOST model was used to define evidence-based questions. P (Population): All pregnant women at potential risk of PPH.I; I (Intervention): Various preventive measures and nursing interventions for primary PPH. P (Professionals): Healthcare professionals (leading roles), patients, and family members. O (Outcome): Incidence of PPH, PPH-related mortality, effective execution rate of emergency protocols, and healthcare professionals’ knowledge acquisition rate of the best evidence. S (Setting): Birthing units at all levels of care in Japan were included. T (Type of evidence): Clinical guidelines, clinical decisions, expert consensus, and evidence summary.

### Evidence retrieval strategy

According to the “6S” pyramid evidence model (6), a top-down systematic search was conducted using computers. We devised a specific search strategy to comprehensively and systematically gather various types of evidence regarding the prevention and management of postpartum hemorrhage. The search began with top-tier authoritative resources and proceeded in a step-by-step and rigorous manner. Multiple internationally recognized and highly influential databases were extensively searched and included, such as, but not limited to, the widely respected clinical decision support tool UpToDate, the evidence-based medicine-focused BMJ Best Practice, the Cochrane Library for systematic reviews, the JBI Evidence-Based Healthcare Center renowned for achievements in evidence-based healthcare, CINAHL with its rich resources in nursing and health sciences, the significant medical literature database Medline, and the biomedical literature database PubMed, among others. These databases have an excellent reputation in the medical field and contain a large volume of rigorously screened and verified high-quality literature, thereby providing a solid data foundation for our research. Additionally, the scope of the literature search covered guideline-issuing organizations from multiple specialties, including the World Health Organization (WHO) and the National Guideline Clearinghouse (NGC) in the United States. The clinical guidelines and related documents published by these organizations represent authoritative perspectives and best practice experiences internationally, offering valuable references for guiding clinical decisions. Simultaneously, recognizing the importance of domestic academic resources, China National Knowledge Infrastructure (CNKI), a core domestic academic database, was included in the search. The search pathway further involved multiple professional society websites, such as the French College of Gynecologists and Obstetricians (CNGOF), Royal College of Obstetricians and Gynecologists (RCOG) in the UK, and the American College of Obstetricians and Gynecologists (ACOG). These social websites bring together the expertise and experience of leading experts in the field, providing clinical decision support, evidence summaries, and consensus statements, all of which are highly instructive for a deeper understanding of the prevention and management of postpartum hemorrhage.

Through this comprehensive and systematic search strategy, we gathered a wide range of evidence, including clinical guidelines, clinical decision documents, evidence summaries, and consensus statements, to provide thorough, scientific, and authoritative references for the prevention and management of postpartum hemorrhage. In the selection process, precisely matched combinations of Chinese and English keywords were used: English keywords included “postpartum hemorrhage/postpartum bleeding,” “obstetric hemorrhage/obstetric bleeding,” “guideline/practice guideline/clinical; decisions/consensus/summary/evidence summary”; while Chinese keywords included “产后出血/产科出血” and “指南/临床实践指南/临床决策/共识.” This ensured that the target types of literature—guidelines, clinical decisions, expert consensus, and evidence summaries—were accurately and comprehensively searched, maximizing the extraction of high-quality evidence closely related to the prevention and management of postpartum hemorrhage.

### Inclusion and exclusion criteria

#### Inclusion criteria

The included studies focused on the prevention and management of postpartum hemorrhage. The study participants were limited to the parturients. The publication language was either Chinese or English. The types of literature must be clinical guidelines published within the past 10 years, decision-making references for clinical practice, expert consensus in the industry, or evidence summaries based on evidence-based medicine.

#### Exclusion criteria

Literature with missing information, leading to a lack of key content, or literature for which the complete original text could not be obtained. Literature published a long time ago is difficult to reflect on the latest research developments and the current clinical practice requirements. Literature that, after a rigorous quality assessment process, is judged to not meet the required standards and cannot provide a reliable basis for the study. Materials that simply offered a literal translation of existing guidelines without innovative interpretation or consideration of local applicability or those that were merely brief summaries of guidelines and failed to cover the core points and detailed information were excluded.

### Literature screening and data extraction

Two systematically trained researchers independently assessed the quality of the different types of literature according to the inclusion and exclusion criteria, and then cross-checked the results. If there was any disagreement between the two researchers, an evidence-based medicine expert was consulted to resolve them. Basic information from the literature was summarized and accurately reviewed.

### Data extraction and error minimization

In order to minimize errors during the data extraction process, two researchers developed a standardized data extraction form *a priori*, based on the JBI data extraction template. The form includes the following fields: author, year of publication, source, type of literature, study population, characteristics of the intervention, outcome measures, and key findings related to PPH prevention. The two researchers independently piloted the form on three randomly selected articles to ensure consistency and clarity, resolving any differences through discussion. Subsequently, each researcher extracted data from half of the included articles, with the other researcher cross-checking the extracted data. Any disagreements were resolved by consulting a third expert. This dual extraction and cross-validation process minimized the risk of omissions or transcription errors.

### Evidence quality evaluation criteria

In the hierarchy of evidence in the medical field, the clinical decision-making UpToDate evidence-based medicine database holds an extremely important position, and is at the top of the evidence pyramid. The evidence provided was of high grade and quality. Researchers in this database conduct in-depth analyses of a vast array of evidence, carefully selecting portions that align with the clinical realities in our country and fully adopting them in their research. The aim of this study was to provide medical practitioners in our country with valuable and applicable evidence-based resources to support the precise formulation of clinical decisions.

Guideline quality evaluation criteria: In this study, the guidelines were evaluated using the “Appraisal of Guidelines for Research & Evaluation Instrument (AGREE II),” which was updated in the UK in 2010 (7) (see Appendix for further details). This scale covers six domains, including 23 specific evaluation items and two overall assessment items. Two researchers independently scored each item on a 7-point scale (where 1 means “strongly disagree” and 7 means “strongly agree”). The standardized percentage for each scoring domain was then calculated using the following formula: standardized percentage = (actual score – minimum possible score) / (maximum possible score – minimum possible score) × 100%. Finally, researchers assign a recommendation Grade A based on the standardized percentage: if the majority of domains score 60.00% or above, it is classified as a Grade A recommendation; if most scores lie between 30 and 60%, it is a Grade B recommendation; and if most scores are below 30%, it is a Grade C recommendation (7).

Quality evaluation criteria for evidence summaries: When assessing the quality of evidence summaries, attention is mainly paid to the original research on which the evidence is based. The Joanna Briggs Institute (JBI) quality assessment tools from Australia, tailored for various types of original research, will be used for the evaluation (8).

For expert consensus assessments, the 2016 evaluation criteria published by the Australian JBI Center for Evidence-Based Healthcare were used (9). These criteria include six evaluation items, and the final evaluation result falls into one of three categories: “Yes,” “No,” or “Unclear.”

When determining the level of evidence and recommendation grade, researchers used the Grading of Recommendations Assessment, Development, and Evaluation (GRADE) system to scientifically analyze the evidence. Assessment of recommendation grade: Strong Recommendation: Clearly indicates that the benefits of the intervention far outweigh the risks, or the risks far outweigh the benefits; therefore, most patients should opt to accept or decline the intervention. Weak Recommendation: The pros and cons of the intervention are uncertain or may vary among patients; therefore, patient preferences and values play an important role in decision-making. If the two researchers reached opposing conclusions during the evidence evaluation process, a third party was introduced to consult and arbitrate. The third party consisted of members of the hospital’s expert panel, specifically an experienced chief obstetrician and a highly skilled associate’s chief physician.

According to the GRADE system, evidence levels are classified as follows: Level 1 (High): High-quality systematic reviews or meta-analyses, high-quality RCTs. Level 2 (Moderate): Low-quality systematic reviews or meta-analyses, low-quality RCTs, or high-quality cohort studies; Level 3 (Low): High-quality case–control studies, low-quality case–control studies; Level 4 (Very Low): Case series or low-quality cohort studies; Level 5 (Very Low): Expert opinion or evidence based on physiology or laboratory research.

Based on the GRADE system, recommendation grades are divided as follows: Grade A (Strong Recommendation) clearly shows that the benefits of the intervention outweigh the harms or vice versa and is applicable to most patients. Grade B (Weak Recommendation): The pros and cons of the intervention are uncertain or may differ among patients, requiring decision-making that considers individual circumstances and values (10).

By adopting the GRADE system, this study scientifically and systematically assessed the quality of evidence, ensuring its reliability and applicability. The use of the GRADE system has improved the transparency and rigor of evidence assessment, providing a solid theoretical foundation for the best evidence summary for the prevention and care of postpartum hemorrhage.

### Assessment of publication bias and conflicts of interest

Regarding the summary of this evidence, publication bias is indirectly assessed through the transparency of conflict of interest disclosures. We used the AGREE II tool to evaluate the quality of the included guidelines, with Domain 6 (“Editorial Independence”) aimed at assessing whether the competing interests of funding bodies or guideline developers have influenced the recommendations. For the included guidelines, two researchers independently evaluated the transparency of conflict of interest statements and funding sources. Guidelines that scored below 50% in Domain 6 were considered to have potential bias and were recorded accordingly. For expert consensus and systematic reviews, assessment was based on relevant items in the JBI quality appraisal checklist regarding whether conflicts of interest were reported. All included literature demonstrated sufficient transparency.

## Results

### General characteristics of the included articles

**Figure 1:**
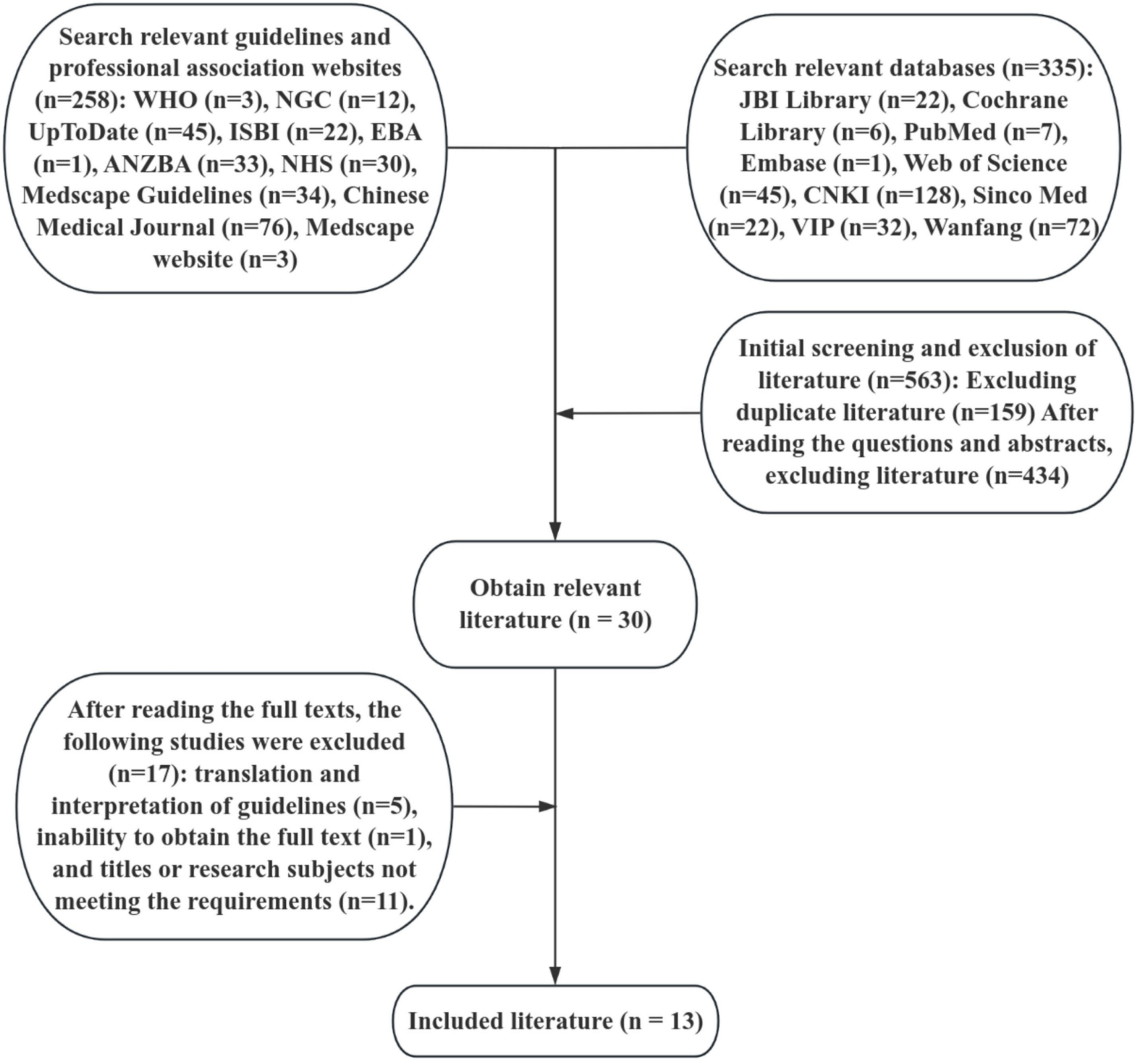
Screening process and results.

The preliminary search yielded 593 relevant articles. After excluding duplicate publications, guideline interpretation articles, and literature unrelated to the research topic, 13 articles were included. Specifically, these 13 articles consisted of two guidelines, two expert consensus statements, one evidence summary, three nursing and survey research articles, one systematic review, and three review articles. The process and results of the literature screening are detailed in Figure 1, and the general characteristics of the included articles are shown in Table 1.

**Table 1:** General characteristics of the included literature (*n* = 13).

Author	Publication years	Document source	Document type	Document theme
Obstetrics Group, Obstetrics and Gynecology Branch of Chinese Medical Association; Perinatal Medicine Branch of Chinese Medical Association (2)	2023	YiigLE	Guideline	Prevention and management of postpartum hemorrhage
Lu et al. (19)	2020	CNKI	Summary of evidence	Prevention and management of postpartum hemorrhage
Chen and Zhang (20)	2024	YiigLE	Clinical decision	Postpartum hemorrhage after vaginal delivery
Li and Chen (21)	2019	CNKI	Nursing research	The application of evidence-based nursing in obstetric clinical practice
Zhou et al. (22)	2024	CNKI	Nursing research	Quality evaluation of textual evidence in the JBI Evidence-Based Healthcare Center
Obstetrics Group, Obstetrics and Gynecology Branch, Chinese Medical Association (11)	2014	Wanfang Data	Guideline	Prevention and management of postpartum hemorrhage
Liu and Yang (23)	2013	YiigLE	Review	Evaluation of prevention and treatment measures for postpartum hemorrhage
Zhang (24)	2017	YiigLE	Short treatise	Analysis of high-risk factors for postpartum hemorrhage
Belfort et al. (25)	2024	UpToDate	Literature review	Postpartum hemorrhage
Sentilhes et al. (26)	2016	PubMed	Expert consensus	Postpartum hemorrhage: Prevention and treatment
Hofer et al. (27)	2023	PubMed	Expert consensus	Hemostatic support in postpartum hemorrhage
Ranjbar et al. (28)	2023	BMJ	Systematic review	Predict the risk of postpartum hemorrhage using machine learning methods
Alonso-Burgos et al. (29)	2024	Embase	Literature review	Primary and secondary postpartum hemorrhage

### Quality evaluation results of the included articles

#### Quality evaluation results of the guidelines

This study included two guidelines (2, 11) obtained from the Chinese Medical Association YiigLE and Wanfang Medical Network. Both guidelines were evaluated using the AGREE II instrument (9). The standardized scores and evaluation results for each domain of the guidelines are listed in Table 2.

**Table 2:** Results of quality evaluation of the clinical guidelines.

Included literature	Percentage of standardization in each field (%)						More than 30% of the fields	More than 60% of the fields	Level recommendation
	Scope and purpose	Involved personnel	The rigor of guideline development	The clarity presented in the guide	The applicability of the guideline	The independence of guideline compilation			
Obstetrics Group, Obstetrics and Gynecology Branch, Chinese Medical Association (2014 Edition) (11)	50.00	41.67	39.58	66.67	45.83	83.33	2	6	B
Obstetrics Group, Obstetrics and Gynecology Branch of Chinese Medical Association; Perinatal Medicine Branch of Chinese Medical Association (2023 Edition) (2)	50.00	41.67	50.00	83.33	66.67	83.33	0	8	A

#### Quality evaluation results of the expert consensus

The expert consensus included in this study was evaluated according to the Australian JBI Expert Consensus Quality Appraisal Checklist (2016). All evaluation items were rated as “Yes,” indicating that the research topic was clear, the approach was well-defined, and the quality of the literature was high.

#### Quality evaluation results of the systematic review

This study included systematic reviews sourced from BMJ, all of which were rated as “Yes” and were therefore included.

#### Quality evaluation results of the clinical decisions

Eight clinical guidelines were included in this study, sourced from CNKI, YiigLE, Embase, and UpToDate. After assessment, their overall quality was found to be high, and they were approved for inclusion.

#### Quality evaluation results of the evidence summaries

A review of the included literature resulted in the identification of the 23 best pieces of evidence, covering 11 aspects: risk assessment, anemia management, health education, choice of delivery method, application of oxytocin, active management of the third stage of labor, control of the labor process, monitoring of vital signs, monitoring of vaginal blood loss, monitoring of uterine contractions, and psychological care. This evidence provides clear guidance for clinical medical staff and helps reduce the incidence and mortality of postpartum hemorrhage. A summary of the best evidence for the prevention and management of postpartum hemorrhage (PPH) is shown in Table 3.

**Table 3:** Summary of best evidence for the prevention and management of PPH in postpartum hemorrhage.

Project	Content of evidence	Evidence level	Recommendation Intensity
Risk assessment	All pregnant women should undergo a comprehensive assessment of the risk of postpartum hemorrhage during their first prenatal visit, including past medical history (such as multiple miscarriages, placenta previa, uterine fibroids, etc.), pregnancy complications (such as gestational hypertension, gestational diabetes, etc.), current pregnancy status (such as multiple pregnancies, macrosomia, etc.), maternal age, weight, and other factors	Level 2	B
	For pregnant women with high-risk factors, reassessment should be conducted in the late stage of pregnancy, with enhanced management and the development of an individualized delivery plan	Level 2	B
Anemia management	During pregnancy, regular blood tests should be conducted to detect and correct anemia in a timely manner. For iron-deficiency anemia, iron supplements and vitamin C can be administered to improve hemoglobin levels in pregnant women	Level 1	A
	It is recommended that hemoglobin levels before delivery should reach 110 g/L or above to enhance pregnant women’s tolerance to blood loss	Level 2	B
Health education	Provide prenatal health education to pregnant women and their families, including knowledge of the causes, symptoms, and preventive measures of postpartum hemorrhage, to increase their awareness and understanding of postpartum hemorrhage	Level 2	B
	Guided pregnant women in prenatal care, proper diet, appropriate exercise, and maintaining a positive mindset to promote physical and mental well-being	Level 2	B
The choice of mode of delivery	According to the specific circumstances of the pregnant woman, such as fetal size, fetal position, pelvic condition, and mother’s wishes, a suitable method of delivery should be chosen. For pregnant women with indications for cesarean section, the procedure should be performed in a timely manner; however, unnecessary cesarean sections should be avoided	Level 1	A
	For pregnant women undergoing vaginal delivery, thorough preparation for childbirth should be made, including monitoring and managing the stages of labor, minimizing the duration of the second stage as much as possible, and preventing excessive maternal fatigue	Level 2	B
The application of oxytocin	After delivery of the fetal anterior shoulder, oxytocin should be administered prophylactically to promote uterine contraction and reduce postpartum hemorrhage. The common routes of administration of oxytocin are intravenous drip or intramuscular injection, with a dosage of 10–20 units	Level 1	A
	For women undergoing cesarean section, 10–20 units of oxytocin can be injected into the uterine body immediately after delivery of the fetus, while an intravenous drip of oxytocin is administered to maintain uterine contractions	Level 1	A
Actively handle the third stage of labor	After the fetus is delivered, the umbilical cord should be gently pulled within 1–2 min to assist with the delivery of the placenta, but excessive force should be avoided to prevent retained placenta or uterine inversion	Level 2	B
	After delivery of the placenta, it is necessary to carefully and thoroughly examine the placenta and fetal membranes for completeness. If any remnants are found, uterine evacuation should be promptly performed	Level 1	A
	Uterine massaging is an important measure to promote uterine contractions and prevent postpartum hemorrhage. After delivery of the placenta, continuous uterine massage should be started immediately and continued until the uterus contracts well and vaginal bleeding is reduced	Level 1	A
Control the labor process	Closely monitor the progress of labor to avoid it being excessively prolonged or too rapid. For women in labor with inadequate uterine contractions and after ruling out cephalopelvic disproportion, oxytocic agents may be appropriately used to strengthen contractions	Level 2	B
	For women experiencing precipitous labor, preparations should be made to prevent postpartum hemorrhage, such as establishing intravenous access in advance and preparing medications like oxytocin	Level 2	B
Monitoring of vital signs	After delivery, the mother’s vital signs, including blood pressure, heart rate, respiration, and temperature, should be closely monitored every 15 to 30 min until they are stable	Level 1	A
	The mother’s complexion, consciousness, skin temperature, and humidity were observed to assess her blood loss	Level 2	B
Vaginal bleeding volume monitoring	Accurate measurement of postpartum vaginal bleeding is key to the early detection of postpartum hemorrhage. Methods such as gravimetric, volumetric, or area methods can be used for measurement	Level 1	A
	Postpartum hemorrhage is most likely to occur. Special attention should be paid to vaginal bleeding in mothers. If an increase in vaginal bleeding is observed, the cause should be promptly identified, and appropriate measures should be taken	Level 1	A
Monitoring of uterine contractions	Uterine contractions, including the height and firmness of the uterine fundus, were regularly monitored. If weak uterine contractions are detected, uterine massage should be intensified, and uterotonic agents should be used as prescribed by a physician	Level 1	A
	Guide mothers to proper breastfeeding. When the baby suckles at the nipple, it reflexively triggers uterine contractions and reduces postpartum bleeding	Level 2	B
Psychological care	Women who experience postpartum hemorrhage often develop negative emotions, such as anxiety and fear, which may further affect uterine contractions and increase bleeding. Therefore, healthcare staff should show care and provide comfort to these women, explain their condition and treatment measures, help alleviate their anxiety, and strengthen their confidence in overcoming their illness	Level 2	B
	Encourage family members to accompany the mother, provide emotional support, and work together to promote recovery	Level 2	B

## Discussion

### Prenatal prevention

Postpartum hemorrhage (PPH) is the leading cause of maternal mortality. Assessment of its risk factors, early prediction, accurate estimation of blood loss, and advance preparation of preventive and treatment measures are crucial for enabling medical teams to implement timely and effective interventions to stop bleeding. According to multiple international guidelines and the consensus of the Network for Advancement of Transfusion Alternatives (NATA) (12), high-risk factors for postpartum hemorrhage have been systematically identified and clarified. In clinical practice, special attention should be paid to these factors during assessments to take preventive measures and reduce the incidence of postpartum hemorrhage. These factors include multiple pregnancies (13), a history of postpartum hemorrhage, chorioamnionitis, assisted vaginal delivery, cesarean section, delivery of a macrosomic infant (13, 14), advanced maternal age (usually over 35 years), obesity (14), anemia (15), conception through assisted reproductive technologies (16), gestational diabetes (17), labor induction (16), prolonged second stage of labor (14), and prolonged third stage of labor (13). These factors not only increase the risk of postpartum hemorrhage but may also impact the severity of bleeding and prognosis.

Currently, in clinical practice in our country, an inaccurate estimation of postpartum blood loss is a common issue. Solely relying on laboratory indicators or the mother’s vital signs to assess postpartum blood loss presents a lag, making it difficult to accurately reflect the actual bleeding situation of the mother in real time. Relevant evidence suggests that more objective and accurate methods, such as the gravimetric (weighing) method or volumetric method, should be adopted in clinical practice to assess postpartum blood loss. Close monitoring of dynamic changes in the mother’s vital signs and paying close attention to the rate of blood loss are of utmost importance to prevent delayed rescue due to the underestimation of postpartum bleeding, thereby effectively avoiding varied degrees of postpartum hemorrhage and even life-threatening consequences. Risk Assessment: In obstetric medical services, to ensure the safety and health of pregnant women during childbirth, it is essential to conduct a comprehensive and meticulous postpartum hemorrhage risk assessment for every pregnant woman during her first prenatal checkup. This assessment needs to consider multiple factors, including the woman’s medical history. For example, a history of multiple miscarriages can damage the endometrium and increase the risk of uterine atony after delivery; placenta previa may lead to abnormal placental separation during labor, resulting in massive bleeding; and uterine fibroids may affect the normal contraction patterns and function of the uterus. Complications arising during pregnancy are also critical for evaluation. Conditions such as gestational hypertension can cause vascular spasms, compromising uteroplacental blood circulation and thereby increasing the risk of postpartum hemorrhage. Gestational diabetes may cause fetal developmental abnormalities such as macrosomia, which can make the delivery process more difficult and increase the likelihood of postpartum bleeding. The specific circumstances of the current pregnancy, such as multiple pregnancies leading to excessive uterine expansion, slow postpartum recovery, and predisposition to atonic bleeding, must also be considered. Factors such as the mother’s age and weight are equally important; advanced maternal age is associated with diminished physical function and reduced recovery abilities from birth trauma, while abnormal weight (being either overweight or underweight) may also adversely affect delivery.

For pregnant women identified as having high-risk factors through assessment, a detailed risk evaluation should be repeated during later stages of pregnancy. The subsequent assessment at this stage enabled a more accurate understanding of the mother’s physical condition and potential risks as a delivery approach. For these high-risk women, prenatal management measures should be strengthened with an increased frequency and range of prenatal examinations and close monitoring of both maternal and fetal physiological parameters. Based on an individual’s unique characteristics, a personalized delivery plan should be developed, fully considering all possible scenarios and making contingency plans in advance to minimize the risk of postpartum hemorrhage to the greatest extent possible (18).

The standardized management of anemia during pregnancy is an important aspect of perinatal care, with its core focus on establishing a systematic monitoring and intervention system. Through regular complete blood count screenings, healthcare providers can promptly identify anemia in pregnant women and adopt targeted treatment measures based on specific causes. In clinical practice, iron deficiency anemia (IDA) is the most common type of anemia during pregnancy, occurring in approximately 15–25% of cases. For pregnant women diagnosed with IDA, a combined supplementation approach is recommended: daily oral iron supplements (such as ferrous sulfate, 300 mg) paired with vitamin C (200 mg). This combined medication regimen has a significant synergistic effect, as vitamin C acts as a reducing agent, converting ferric iron to more easily absorbable ferrous iron, and also promotes the synthesis of ferritin, enhancing iron bioavailability by 2–3 times. Clinical studies indicate that standardized iron supplementation can increase hemoglobin by approximately 1 g/dL per week. To ensure safe delivery, the World Health Organization (WHO) recommends maintaining hemoglobin levels at ≥110 g/L during late pregnancy. This standard, which is based on evidence-based medicine, significantly improves a pregnant woman’s tolerance for blood loss during delivery. Adequate hemoglobin levels can keep tissue oxygenation indices within the normal range (PaO2 80–100 mmHg), ensuring oxygen delivery in the placental-fetal circulation and reducing risk factors for fetal distress and postpartum hemorrhage. Building and implementing a prenatal health education system is a core element of perinatal care quality management. Based on evidence-based medical concepts, it is recommended to establish a multidimensional, stratified health education model targeting pregnant women and their primary caregivers (such as spouses and immediate family members). Educational content should follow systematic and standardized principles, with a focus on explaining postpartum hemorrhage (PPH), an obstetric emergency. Key points for the clinical recognition of PPH include: when vaginal bleeding is ≥500 mL (≥1,000 mL for cesarean delivery) within 24 h postpartum, or there is a progressive drop in hemoglobin (>20 g/L), PPH should be suspected. Typical clinical manifestations include: (1) Bleeding signs: persistent vaginal bleeding or expulsion of blood clots; (2) Shock symptoms: pale complexion, cold and clammy skin, pulse >110 beats/min, systolic blood pressure < 90 mmHg; (3) Inadequate tissue perfusion: urine output <30 mL/h, altered consciousness, etc.

### Intrapartum prevention

Evidence-based Medical Practice in Delivery Decision-making: Based on the principles of individualized medicine, the choice of delivery method should be based on a comprehensive assessment of the maternal-fetal system. Key assessment parameters include Estimated Fetal Weight (EFW), fetal position (such as cephalic or breech presentation), pelvic morphology measurements (including diagonal conjugate and ischial spine distance), and the mother’s psychosocial factors. For cases that meet the indications for cesarean section (such as placenta previa and fetal distress), surgical intervention should be promptly performed to optimize maternal and neonatal outcomes. At the same time, the WHO-recommended standards must be strictly followed, keeping the cesarean section rate for non-medical reasons within a reasonable range (recommended ≤ 15%) to reduce the risk of surgery-related complications (such as postpartum hemorrhage, infection, thrombosis, etc.). A comprehensive management model should be implemented for women assessed as suitable for vaginal deliveries. Key points include: (1) Establishing an individualized labor monitoring plan and using a partogram for dynamic monitoring; (2) Optimizing second-stage management, recommending it be limited to within 1 h for multiparas and 2 h for primiparas; and (3) Implementing preventive interventions, such as free positioning during delivery and perineal protection techniques, to reduce the risk of perineal injury and postpartum hemorrhage. Use and Management of Oxytocin: Based on evidence-based medical data, it is recommended to initiate prophylactic medication immediately after delivery of the anterior shoulder of the fetus. The standard administration protocol is intravenous injection of 10 U (diluted in 10 mL saline) or intramuscular injection of 10 U. For cesarean section cases, a combined administration protocol is recommended: direct injection into the uterine body of 10–20 U along with intravenous infusion of 10–20 U (added to 500 mL crystalloid solution, at 20–40 drops per minute), to maintain effective uterine contractions. Management of the Third Stage of Labor: Controlled Cord Traction (CCT) is recommended within 1–2 min after delivery of the fetus to avoid iatrogenic complications. After placental delivery, a systematic examination should be conducted to confirm placental integrity (maternal surface, fetal surface, and marginal vessels), measure placental diameter and thickness, and assess the membrane integrity. If retention is observed, timely ultrasound-guided curettage should be performed. Uterine massage: Use the bimanual massage method, maintaining a frequency of 15–20 times per minute, with the firmness judged by the uterus becoming noticeably hard. The duration of massage should be dynamically adjusted according to the strength of uterine contractions and the volume of bleeding, usually lasting 15–30 min. Precision Strategies for Labor Management: It Electronic fetal heart monitoring combined with uterine contraction pressure monitoring is recommended to establish an early warning mechanism. For cases with a tendency toward precipitous labor, a rapid response team should be established to ensure that intravenous access is unobstructed and to prepare emergency medications (such as carbetocin and misoprostol) to build a comprehensive safety assurance system.

### Postpartum prevention

Severe postpartum hemorrhage is a critical condition in obstetrics, and it is difficult for obstetricians to effectively manage it. During pregnancy, the physiological state of a woman is unique. Her vital organs are constantly under high-load operation, and her coagulation function is also in a delicate balance between the excessive activation of procoagulants and anticoagulants. Under such a fragile physiological state, once the amount of bleeding increases rapidly, it is highly likely to cause multiple system and organ failure within a short period, seriously threatening the safety of the parturient. Therefore, it is very important to form a multidisciplinary joint rescue team. The team should include experienced obstetricians, professional midwives and nurses, anesthesiologists, gynecologists, hematologists, critical care physicians, and interventional radiologists. Members of each discipline in the team need to be clear about their own responsibilities, work closely together, properly handle the relationship between the part and the whole, ensure that rescue work proceeds efficiently and orderly, and win a precious opportunity to save the life of the parturient.

The most common cause of postpartum hemorrhage is uterine atony, which accounts for approximately 60–70% of all postpartum hemorrhage cases. However, the underlying mechanism of uterine atony remains unclear, which has become a key obstacle restricting further development of predictive methods and prevention strategies for postpartum hemorrhage. Oxytocin is the first-line drug for the treatment of postpartum hemorrhage caused by uterine atony. However, if oxytocin does not achieve the expected hemostatic effect, it is necessary to promptly switch to other uterotonic agents, with ergometrine being a commonly used alternative. Regardless of the cause of postpartum hemorrhage, once diagnosed, tranexamic acid should be administered as early as possible. As an antifibrinolytic drug, tranexamic acid has shown significant effects in the treatment of postpartum hemorrhage and is suitable for patients with postpartum hemorrhage of various etiologies. If uterotonic agents fail to effectively stop bleeding, other methods of hemostasis must be quickly sought, including intrauterine tamponade and other surgical hemostatic procedures. Uterine massage is a commonly used and effective clinical method for promoting uterine contractions. Bleeding can be reduced by stimulating the uterus. When symptoms of postpartum hemorrhage cannot be effectively controlled using uterotonic agents and uterine massage alone, the condition is considered refractory postpartum hemorrhage. In cases of postpartum hemorrhage following vaginal delivery, intrauterine tamponade is often the preferred hemostatic measure if uterine atony-related bleeding does not respond to massage or medication.

### Limitations of evidence in this study

First, although a comprehensive search strategy was designed based on the “6S” pyramid model, the search was limited to Chinese and English literature, which may introduce language bias and potentially exclude relevant evidence published in other languages. Second, the search dates for each database and website were not explicitly reported, which limits the reproducibility and timeliness of the search process. Third, although literature screening and quality assessment were conducted independently by two researchers, a formal protocol was not registered before carrying out the review, which may increase the risk of reporting bias. Fourth, the quality assessment of the included studies relied on various evaluation tools (such as AGREE II, JBI checklists, etc.); although cross-checking was performed, the subjectivity of these tools may introduce a certain degree of variability in the assessment. Finally, the details and reasons for excluding studies were not sufficiently reported, limiting the transparency of the study selection process. Future updates of this evidence summary should consider protocol registration, expanding the language range, and more clearly documenting the screening and exclusion process to enhance methodological rigor and transparency.

### Future research directions

Although this study has comprehensively synthesized the best evidence for the prevention and management of postpartum hemorrhage (PPH), several knowledge gaps and methodological limitations highlight the need for further research. First, most of the included evidence comes from international guidelines and expert consensus, with a lack of high-quality original studies conducted in low- and middle-income regions, where the burden of PPH is greatest. Future research should prioritize large-scale, multicenter prospective cohort studies and randomized controlled trials in these settings to verify the applicability and effectiveness of existing evidence. Second, although the GRADE system was used to assess the quality of evidence, many recommendations are supported by moderate or low levels of evidence, indicating a need for high-quality randomized controlled trials to address key clinical questions. Third, implementation science research is needed to identify the barriers and facilitators to adopting evidence-based PPH protocols in real-world clinical settings, especially in resource-limited environments. Fourth, future evidence syntheses and guidelines should take into account patient-reported outcomes and preferences, including the psychological impact of PPH and satisfaction with care, areas that remain insufficiently explored. Qualitative research can enrich our understanding of maternal experiences and inform more comprehensive models of care. Addressing these research priorities will strengthen the evidence base for PPH prevention and management, ultimately helping to reduce maternal morbidity and mortality worldwide.

### Summary

This study comprehensively reviews the best evidence for the prevention and management of postpartum hemorrhage, summarizing it across the prenatal, intrapartum, and postpartum periods in 11 dimensions: risk assessment, anemia management, health education, mode of delivery selection, use of oxytocin, active management of the third stage of labor, labor process control, monitoring of vital signs, monitoring of vaginal blood loss, monitoring of uterine contractions, and psychological care. This achievement provides a solid theoretical foundation for clinical birthing units and healthcare professionals in the prevention and management of postpartum hemorrhage. It is noteworthy that most of the evidence included in this study was derived from international clinical guidelines. When applying the best evidence summarized in this research, each birthing unit should fully respect the opinions of clinical managers, comprehensively assess their own conditions, cultural context, possible barriers, and facilitators in the process of evidence implementation, and place a high value on patient preferences. In combination with the current realities of medical practice in China, these pieces of evidence should undergo localization and adaptation to ensure their smooth application in clinical practice, thereby effectively improving PPH prevention and management of postpartum hemorrhage.

## Data Availability

The raw data supporting the conclusions of this article will be made available by the authors, without undue reservation.
